# Update: Severe Respiratory Illness Associated with a Novel Coronavirus — Worldwide, 2012–2013

**Published:** 2013-03-15

**Authors:** Div of Viral Diseases, National Center for Immunization and Respiratory Diseases, Brian Rha

**Affiliations:** EIS Officer, CDC

CDC continues to work closely with the World Health Organization (WHO) and other partners to better understand the public health risk posed by a novel coronavirus that was first reported to cause human infection in September 2012 (1–3). Genetic sequence analyses have shown that this new virus is different from any other known human coronaviruses, including the one that caused severe acute respiratory syndrome (SARS) (2). As of March 7, 2013, a total of 14 confirmed cases of novel coronavirus infection have been reported to WHO, with eight deaths (4). Illness onsets have occurred from April 2012 through February 2013 (4,5). To date, no cases have been reported in the United States.

Three of the confirmed cases of novel coronavirus infection were identified in the United Kingdom (UK) as part of a cluster within one family (6). The index patient in the cluster, a man aged 60 years with a history of recent travel to Pakistan and Saudi Arabia, developed respiratory illness on January 24, 2013, before returning to the UK on January 28 (5,7,8). He was hospitalized on January 31 with severe lower respiratory tract disease and has been receiving intensive care (5,7,8). Respiratory specimens from this patient taken on February 1 tested positive for influenza A (H1N1) virus and for novel coronavirus infection (8). The second patient was an adult male household member with an underlying medical condition who became ill on February 6, after contact with the index patient, and received intensive treatment but died with severe respiratory disease (5,9). This patient’s underlying illness might have made him more susceptible to severe respiratory infection. The third patient is an adult female who developed a respiratory illness on February 5, following contact with the index patient after he was hospitalized (5,10). She did not require hospitalization and had recovered by February 19 (5,6). Only the index patient had traveled recently outside the UK. Based on their ongoing investigation of this cluster of illnesses, the UK Health Protection Agency has concluded that person-to-person transmission likely occurred in the UK within this family (6).

This recent cluster provides the first clear evidence of human-to-human transmission of this novel coronavirus, coinfection of this novel coronavirus with another pathogen (influenza A), and a case of mild illness associated with this novel coronavirus infection. In light of these developments, updated guidance has been posted on the CDC coronavirus website (http://www.cdc.gov/coronavirus/ncv). Persons who develop severe acute lower respiratory illness within 10 days after traveling from the Arabian Peninsula or neighboring countries* should continue to be evaluated according to current guidelines. Persons whose respiratory illness remains unexplained and who meet criteria for “patient under investigation” should be reported immediately to CDC through state and local health departments. Persons who develop severe acute lower respiratory illness of known etiology within 10 days after traveling from the Arabian Peninsula or neighboring countries but who do not respond to appropriate therapy may be considered for evaluation for novel coronavirus infection. In addition, persons who develop severe acute lower respiratory illness who are close contacts† of a symptomatic traveler who developed fever and acute respiratory illness within 10 days of traveling from the Arabian Peninsula or neighboring countries may be considered for evaluation for novel coronavirus infection. Testing of specimens for the novel coronavirus will be conducted at CDC.

Recommendations and guidance on case definitions, infection control (including use of personal protective equipment), case investigation, and specimen collection and shipment for testing, are available at the CDC coronavirus website. Additional information and potentially frequent updates will be posted on the CDC coronavirus website. State and local health departments with questions should contact the CDC Emergency Operations Center (770-488-7100).

## Footnotes

* Countries considered to be on or neighboring the Arabian Peninsula include Bahrain, Iraq, Iran, Israel, Jordan, Kuwait, Lebanon, Oman, Palestinian Territories, Qatar, Saudi Arabia, Syria, the United Arab Emirates, and Yemen.

† Defined as 1) any person who provided care for the patient, including a health-care worker or family member, or who had other similarly close physical contact, or 2) any person who stayed at the same place (e.g., lived with or visited) as the patient while the patient was ill.

## References

[1] Danielsson N, Team EIR, Catchpole M (2012). Novel coronavirus associated with severe respiratory disease: case definition and public health measures. Euro Surveill.

[2] Zaki AM, van Boheemen S, Bestebroer TM, Osterhaus AD, Fouchier RA (2012). Isolation of a novel coronavirus from a man with pneumonia in Saudi Arabia. N Engl J Med.

[3] CDC (2012). Severe respiratory illness associated with a novel coronavirus—Saudi Arabia and Qatar, 2012. MMWR.

[4] World Health Organization (2013). Global alert and response (GAR): novel coronavirus infection—update.

[5] European Centre for Disease Prevention and Control (2013). Rapid risk assessment: severe respiratory disease associated with a novel coronavirus.

[6] Health Protection Agency (2013). Update on family cluster of novel coronavirus infection in the UK.

[7] Health Protection Agency (2013). Case of novel coronavirus identified in the UK.

[8] European Centre for Disease Prevention and Control (2013). Epidemiological update: case of severe lower respiratory tract disease associated with a novel coronavirus—February 11, 2013.

[9] Health Protection Agency (2013). Further UK case of novel coronavirus.

[10] Health Protection Agency (2013). Third case of novel coronavirus infection identified in family cluster.

